# Older people, the natural environment and common mental disorders: cross-sectional results from the Cognitive Function and Ageing Study

**DOI:** 10.1136/bmjopen-2015-007936

**Published:** 2015-09-16

**Authors:** Yu-Tzu Wu, A Matthew Prina, Andy Jones, Fiona E Matthews, Carol Brayne, MRC CFAS

**Affiliations:** 1 Department of Public Health and Primary Care, Institute of Public Health, Forvie Site, University of Cambridge, School of Clinical Medicine, Cambridge, UK; 2 Health Service and Population Research Department, King's College London, Institute of Psychiatry, Psychology and Neuroscience, Centre for Global Mental Health, London, UK; 3 Norwich Medical School, University of East Anglia, Norwich, Norfolk, UK; 4 MRC Biostatistics Unit, Institute of Public Health, University of Cambridge, Cambridge, UK; 5 Institute of Health and Society, Newcastle University, Newcastle, UK

**Keywords:** EPIDEMIOLOGY, PUBLIC HEALTH, MENTAL HEALTH

## Abstract

**Objectives:**

To explore the hypothesis that higher exposure to natural environments in local areas is associated with a lower odds of depression and anxiety in later life.

**Design:**

A cross-sectional study based on the year-10 interview of the Medical Research Council Cognitive Function and Ageing Study (CFAS), a population-based study of ageing in the UK. Postcodes of the CFAS participants were mapped onto small geographic units, lower-layer super output areas (LSOAs) and linked to environmental data from government databases. The natural environment was characterised as the percentage of green space and private gardens in each LSOA based on the UK Generalised Land Use 2001 Dataset.

**Participants:**

2424 people aged 74 and over in the CFAS year-10 follow-up interview (2001) from 4 English centres (Cambridgeshire, Nottingham, Newcastle and Oxford).

**Main outcome measures:**

Depression and anxiety; clinical and subthreshold cases were identified using the Geriatric Mental State Examination (GMS) package and its associated diagnostic algorithm: the Automated Geriatric Examination for Computer Assisted Taxonomy.

**Results:**

Compared with the lowest quartile, living in the highest quartile of neighbourhood natural environment provision was associated with a reduced odds of subthreshold depression (OR 0.66, 95% CI 0.46 to 0.95), anxiety symptoms (OR 0.62, 95% CI 0.46 to 0.83) and their co-occurrence (OR 0.55, 95% CI 0.35 to 0.84) after adjusting for individual-level factors. Controlling for area deprivation attenuated the strength of associations for subthreshold depression by 20% but not for anxiety symptoms or for co-occurrence of the conditions.

**Conclusions:**

A high exposure to natural environments (green space and gardens) in communities was associated with fewer mental disorders among older people. Increasing provision of green environments in local areas could be a potential population-level intervention to improve mental health among older people.

**Strengths and limitations of this study**

- This study was based on a longitudinal population-based cohort of older people, providing detailed assessments of health conditions and mental status in later life.
- The association between natural environments and common mental disorders was investigated using consistent outcome measures with known clinical significance, coupled with objectively defined environmental exposure estimates, in a large sample of older people across England.
- This study was cross-sectional and therefore the associations observed cannot be assumed to be causal.
- The study population was survivors from the baseline interview 10 years earlier and the problem of dropout might potentially have caused selection bias.

## Introduction

Theoretical models suggest that a good exposure to green space, areas with natural vegetation such as grass, trees and plants, will not only encourage physical activity, a protective factor against mental disorders, but can also act as a buffer to psychological distress and deprivation.^1^ Recent reviews have particularly emphasised the beneficial influence of urban green space, including public parks and domestic gardens, on the physical and mental health of the population.^2^ ^3^ There is a particular interest in elements of the natural environment that might mitigate the potential negative influence of adverse environmental characteristics experienced by urban populations, such as overcrowding, pollution and noise.^4^ ^5^

Accordingly, a relationship between mental illnesses and neighbourhood green space has been previously reported.^6–8^ Recent studies in the UK have shown both cross-sectional and longitudinal associations between mental health and green space among younger adults.^9–11^ Although the literature has suggested a positive influence of natural environments on individual mental health, the associations may vary across different age groups.^12^ Few studies have explored these associations in older people who have reduced levels of physical function, a lower pattern of outdoor activity and consequently different levels of exposure to the natural environment compared with their younger counterparts.^13^ Some existing studies have suggested that access to green space is important for age-friendly environments.^14^ ^15^ There has been little research on how the physical environment, and specifically access to green space, may support mental health and well-being among older adults and therefore might be a modifiable influence at the population level. A UK-based study of people aged 65 or above reported that perceived neighbourhood pleasantness, a measure which included the availability of trees and plants, was associated with overall life satisfaction.^16^ However, the sample size was small (n=271) and measure of exposure to green space was based on self-report. A negative relationship between greenness in local areas and psychological distress of older adults has also been reported in recent studies in South Wales, UK and Australia.^6^ ^17^ These studies measured the proportion of green space in neighbourhoods using satellite images or government databases and found that high exposure to green space in local areas was associated with a 10–20% lower odds of psychological distress. However, the association between natural environments, depression and anxiety, important mental health problems with practical and clinical significance, has not been investigated in older age groups. Using data from a large cohort of older people in England, this study explores the association between area-based exposure to the natural environment, measured according to the objectively defined neighbourhood green space and private gardens, and common mental disorders, based on consistent outcome measures of depression and anxiety symptoms.

## Methods

### Study population

The Medical Research Council Cognitive Function and Ageing Study (CFAS) is a longitudinal population-based study investigating cognitive and physical decline of people aged 65 years and over in six centres across England and Wales (Liverpool, Cambridgeshire, Gwynedd, Newcastle upon Tyne, Nottingham and Oxford).^18^ Identical study design and measurement methods were used at each centre except Liverpool, which has been excluded from this analysis.

Full details of CFAS have been described elsewhere.^18^ In brief, community and institutionalised populations were sampled from primary care registrations in order to capture equal sized samples of individuals aged 65–74 and 75 years and over. Baseline interviews were conducted between 1991 and 1994 and delivered by trained interviewers visiting the participants’ home residence. Among 16 258 individuals invited for the study, 13 004 completed the initial screening interview with a response rate of 80%. The main follow-up waves included 1-year follow-up and a 2-year rescreen, new selection for assessment and further a 1-year follow-up, a 6-year follow-up of the assessed, an 8-year follow-up of a specific subgroup and a 10-year follow-up of the whole sample. The analysis presented here focuses on the 2424 participants who attended the year-10 interview in 2001 from the four English centres (Cambridgeshire, Newcastle upon Tyne, Nottingham and Oxford). The centre in Wales (Gwynedd) was excluded due to the lack of comparable information on area deprivation.

For the purposes of this study, a range of variables were extracted from the CFAS:

#### Mental disorders

Depression and anxiety symptoms were measured by the Geriatric Mental State Examination (GMS) and its associated diagnostic algorithm: the Automated Geriatric Examination for Computer Assisted Taxonomy (AGECAT).^19^ A clinical case of depression was defined as an AGECAT depression level of three or above (out of a maximum of five) while a score of one or two was considered a subthreshold case.^19^ Since the number of participants with clinical anxiety (anxiety level 3 and above) was small (N=46), the measure of anxiety used identified all study participants with any anxiety symptoms, which was defined as anxiety level of one or above. Those who had both depression and anxiety symptoms were considered to have co-occurrence of depression and anxiety.

#### Individual-level covariates

Sociodemographic information, including age, gender, education and social class were recorded at the interview. Based on the previous studies from the CFAS baseline, these factors were known to be significantly related to depression and anxiety symptoms in later life.^20^ ^21^ Education was divided into two groups separating people with nine or fewer years of education from those with 10 years and above. The longest occupation reported was used to classify the social class of each participant according to the Registrar General's occupation-based social class tables.^22^ Participants with social class classifications I to IIINM were grouped as the ‘non-manual’ group while those in social class IIIM to V formed the ‘manual’ group. The interview question ‘Have you moved in the last 2 years?’ was used to identify recently relocated participants.

Comorbidity associated with the presence of chronic conditions is known to be an important risk for poor mental health.^23^ The number of chronic illnesses associated with each participant, including hypertension, diabetes, stroke, heart attack, angina, low blood pressure/dizzy on standing, hearing and vision impairment, were recorded based on self-reported information in the year-10 interview and were used in this analysis.

### Community-level measurements: area deprivation and the natural environment

Based on information from the National Statistics Postcode Directory (NSPD), the postcodes of the year-10 participants were mapped to lower-layer super output areas (LSOA), a geographic unit developed for the collation of small area statistics following the 2001 UK Census, with an average of 1500 residents per unit.^24^ In cases where postcodes from the year-10 interview were missing, incomplete or incorrect, the full address was used to obtain complete postcodes from the Royal Mail, Google Maps and property websites.

Environmental data for each LSOA were obtained from the published UK Government Neighbourhood Statistics, a collection of small area-level data across England.^25^ The measure of natural environment exposure employed was the percentage of green space and private gardens in each LSOA based on the Generalised Land Use 2001 Dataset (GLUD), which provides areas of different types of land use in thousands of square metres for all the LSOAs across England (data.gov.uk/dataset/land_use_statistics_generalised_land_use_database). Green space was defined as areas covered with grass and private gardens were grounds adjacent to houses. The data were originally derived from the UK Ordnance Survey MasterMap product, which consists of a series of 1:1250 to 1:10 000 scale maps produced from on-ground surveys (http://www.ordnancesurvey.co.uk/business-and-government/products/mastermap-products.html).

Area deprivation was measured by the English Index of Multiple Deprivation (IMD 2004), which was based on data collected in 2001 and 2002.^26^ The IMD score summarised seven domains of characteristics related to deprivation including income, employment, education and training, health and disability, barriers to housing and services, the living environment and crime.

### Analysis strategy

Two-level multilevel logistic regression, with individuals at level 1 and LSOAs of residence at level 2, was used to explore the association between community-level measurements (area deprivation and natural environment) and the existence of mental disorders. Three types of regression model were fitted. First was a univariable model (model 1) including just one individual-level or community-level factor at a time which was used to investigate their unadjusted associations with depression (clinical or subthreshold cases), anxiety symptoms and their co-occurrence.

The second model (model 2) focused on the association between the natural environment and mental disorders in later life adjusting for individual sociodemographic characteristics and the measure of comorbidity. Model 3 further adjusted for area deprivation to control for the potential influence of other correlated but unmeasured social and environmental factors. It allowed an examination of whether the strength and direction of associations between the natural environment, depression and anxiety changed considerably after full adjustment. Since those who had recently relocated would have had little exposure to the natural environment in their local areas, a sensitivity analysis was carried out by excluding those who reported moving into the areas in the past 2 years. A test for trend was applied to examine whether a linear trend in the odds of mental disorders was present across quartiles of neighbourhood natural environment exposure. Statistical analyses were performed using Stata V.10.0.

## Results

### Descriptive analysis and individual-level factors

The median age of the 2424 participants was 81 years with a range from 74 to 101. Approximately 60% were women. Most had education of less than 9 years duration (60.1%) and a manual occupation (53.8%). Over 70% reported at least one chronic condition in the interview.

**Figure 1:**
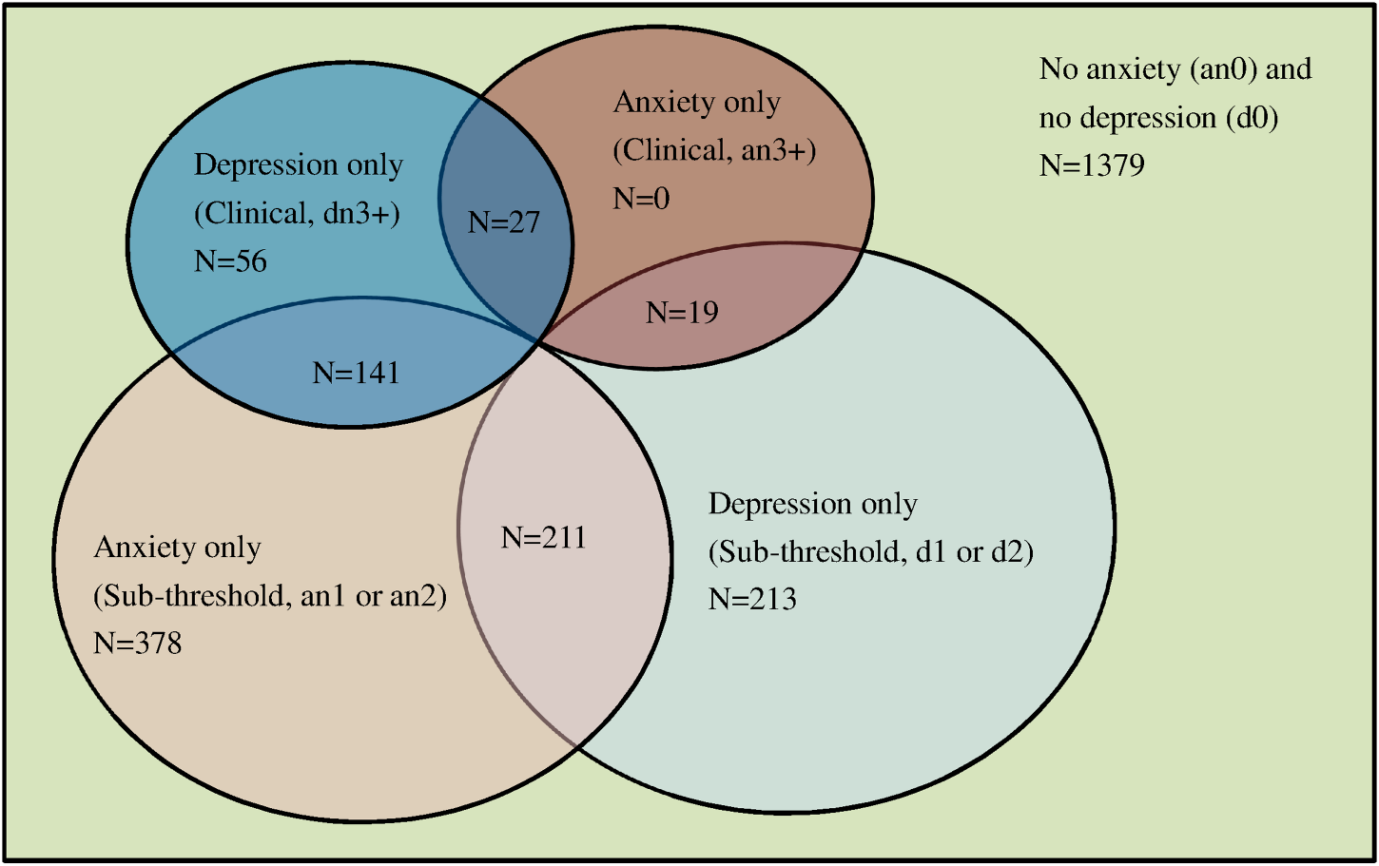
Mix of depression and anxiety symptoms in the sample.

The crude prevalence of clinical and subthreshold level depression was 9.2% and 18.3%, respectively, and over 30% of participants had anxiety symptoms. The co-occurrence of depression and anxiety disorders is graphically presented in figure 1. In total, 398 (16.4%) participants had a co-occurrence of depression and anxiety symptoms. Among the 224 participants with clinical depression, 75% also had clinical (N=27) or subthreshold (N=141) level symptoms of anxiety. In the subthreshold cases of depression, 52% (N=230) had a co-occurrence of anxiety symptoms.

The prevalence of mental disorders according to characteristics of the study population is reported in table 1. The prevalence of depression and anxiety disorders was not higher in the older age groups. A higher proportion of mental disorders presented among women, those of low education and low social class. Women were almost twice likely to have clinical depression than men. A higher number of chronic illnesses was also associated with a higher likelihood of mental disorders; those reporting at least two chronic conditions were between 60% and 80% more likely to have depression or anxiety symptoms compared with those with no chronic conditions.

**Table 1:** The distributions of depression and anxiety disorders across individual-level factors

	Depression symptoms		Anxiety symptoms
	Subthreshold (d1 or d2, %)	Clinical (dn3+, %)	Any level (an1–an5, %)
Total	443 (18.3)	2240 (9.2)	776 (32.0)
Age			
74–79	171 (17.2)	760 (7.7)	304 (30.6)
80–84	144 (18.6)	80 (10.3)	255 (32.8)
85–89	90 (20.5)	390 (8.9)	130 (29.6)
90+	38 (17.5)	5 (13.4)	87 (40.1)
Sex			
Men	137 (14.4)	550 (5.8)	271 (28.4)
Women	306 (20.8)	169 (11.5)	505 (34.3)
Education (years)			
>9	162 (16.8)	710 (7.4)	275 (28.5)
≤9	281 (19.4)	152 (10.5)	498 (34.3)
Social class			
Non-manual	192 (17.3)	930 (8.4)	338 (30.4)
Manual	249 (19.2)	1280 (9.9)	431 (33.3)
Number of chronic illness			
None	100 (14.8)	520 (7.7)	162 (24.0)
One	137 (17.2)	660 (8.3)	267 (33.4)
Two and more	206 (21.7)	106 (11.2)	347 (36.6)

### The natural environment and mental disorders in later life

The modelled associations between the area-based measure of natural environment exposure and mental disorders in later life are reported in tables 2 and 3. Before adjustment (model 1), the lowest odds of reporting all the conditions considered were found in the highest quartile of neighbourhood natural environment exposure, with statistically significant trends across categories of green space for subclinical depression, anxiety and the co-occurrence of symptoms. After adjusting for individual-level factors, living in the highest quartile of natural environment exposure was associated with a 30–40% lower odds of clinical and subthreshold depression and anxiety symptoms (model 2). The decreasing trend in anxiety symptoms achieved statistical significance (p value for trend <0.01). Those living in neighbourhoods with the highest exposure to natural environments had just over half (OR 0.62, 95% CI 0.46 to 0.83) the odds of anxiety symptoms compared with those in the lowest quartile. Excluding those who had moved residence in the past 2 years did not substantially influence the estimates (results not shown).

**Table 2:** The associations between natural environment and depression

	Depression: clinical case			Depression: subthreshold case		
	Model 1 OR (95% CI)	Model 2 OR (95% CI)	Model 3 OR (95% CI)	Model 1 OR (95% CI)	Model 2 OR (95% CI)	Model 3 OR (95% CI)
*Individual-level factors*						
Age	1.03 (1.01 to 1.06)	1.02 (1.00 to 1.05)	1.02 (1.00 to 1.05)	1.01 (0.99 to 1.03)	1.00 (0.98 to 1.02)	1.00 (0.98 to 1.02)
Sex						
Men	1.00 (0.00 to 0.00)	1.00 (0.00 to 0.00)	1.00 (0.00 to 0.00)	1.00 (0.00 to 0.00)	1.00 (0.00 to 0.00)	1.00 (0.00 to 0.00)
Women	2.12 (1.55 to 2.91)	2.11 (1.52 to 2.93)	2.12 (1.53 to 2.94)	1.71 (1.37 to 2.13)	1.74 (1.38 to 2.19)	1.74 (1.38 to 2.19)
Education (years)						
>9	1.00 (0.00 to 0.00)	1.00 (0.00 to 0.00)	1.00 (0.00 to 0.00)	1.00 (0.00 to 0.00)	1.00 (0.00 to 0.00)	1.00 (0.00 to 0.00)
≤9	1.47 (1.10 to 1.98)	1.51 (1.08 to 2.10)	1.47 (1.05 to 2.06)	1.25 (1.01 to 1.55)	1.26 (0.98 to 1.61)	1.16 (0.90 to 1.49)
Social class						
Non-manual	1.00 (0.00 to 0.00)	1.00 (0.00 to 0.00)	1.00 (0.00 to 0.00)	1.00 (0.00 to 0.00)	1.00 (0.00 to 0.00)	1.00 (0.00 to 0.00)
Manual	1.20 (0.91 to 1.59)	1.02 (0.74 to 1.39)	1.00 (0.73 to 1.38)	1.17 (0.95 to 1.44)	1.06 (0.83 to 1.35)	0.99 (0.78 to 1.27)
Number of chronic illness						
None	1.00 (0.00 to 0.00)	1.00 (0.00 to 0.00)	1.00 (0.00 to 0.00)	1.00 (0.00 to 0.00)	1.00 (0.00 to 0.00)	1.00 (0.00 to 0.00)
1	1.08 (0.74 to 1.58)	1.02 (0.69 to 1.51)	1.02 (0.69 to 1.51)	1.20 (0.91 to 1.60)	1.18 (0.88 to 1.58)	1.15 (0.85 to 1.55)
2+	1.51 (1.07 to 2.14)	1.40 (0.97 to 2.01)	1.40 (0.97 to 2.02)	1.69 (1.30 to 2.21)	1.67 (1.26 to 2.21)	1.63 (1.23 to 2.16)
*Area deprivation*						
Q1 (Least)	1.00 (0.00 to 0.00)		1.00 (0.00 to 0.00)	1.00 (0.00 to 0.00)		1.00 (0.00 to 0.00)
Q2	0.95 (0.60 to 1.51)		0.79 (0.49 to 1.27)	1.64 (1.14 to 2.35)		1.52 (1.05 to 2.20)
Q3	1.14 (0.73 to 1.78)		0.94 (0.59 to 1.49)	1.62 (1.13 to 2.32)		1.49 (1.03 to 2.17)
Q4 (Most)	1.44 (0.95 to 2.20)		1.14 (0.70 to 1.84)	2.20 (1.56 to 3.10)		1.98 (1.34 to 2.93)
*Natural environment*						
Q1 (Lowest)	1.00 (0.00 to 0.00)	1.00 (0.00 to 0.00)	1.00 (0.00 to 0.00)	1.00 (0.00 to 0.00)	1.00 (0.00 to 0.00)	1.00 (0.00 to 0.00)
Q2	1.04 (0.69 to 1.55)	0.99 (0.66 to 1.51)	1.01 (0.66 to 1.52)	0.95 (0.69 to 1.31)	0.91 (0.65 to 1.26)	0.97 (0.70 to 1.34)
Q3	0.92 (0.61 to 1.39)	0.97 (0.64 to 1.47)	1.03 (0.67 to 1.59)	0.97 (0.71 to 1.34)	0.98 (0.71 to 1.35)	1.13 (0.81 to 1.58)
Q4 (Highest)	0.67 (0.42 to 1.07)	0.72 (0.45 to 1.16)	0.80 (0.48 to 1.33)	0.64 (0.45 to 0.91)	0.66 (0.46 to 0.95)	0.84 (0.57 to 1.23)
p Value for trend	0.11	0.24	0.52	0.04	0.08	0.68

Model 1: unadjusted; model 2: adjusted for all individual-level factors; model 3: adjusted for all individual-level factors and area deprivation.

**Table 3:** The associations between natural environment, anxiety and co-occurrence of depression and anxiety

	Anxiety symptoms			Co-occurrence of depression and anxiety		
	Model 1 OR (95% CI)	Model 2 OR (95% CI)	Model 3 OR (95% CI)	Model 1 OR (95% CI)	Model 2 OR (95% CI)	Model 3 OR (95% CI)
*Individual-level factors*						
Age	1.01 (0.99 to 1.03)	1.01 (0.99 to 1.02)	1.01 (0.99 to 1.02)	1.01 (0.99 to 1.03)	1.00 (0.98 to 1.03)	1.00 (0.98 to 1.03)
Sex						
Men	1.00 (0.00 to 0.00)	1.00 (0.00 to 0.00)	1.00 (0.00 to 0.00)	1.00 (0.00 to 0.00)	1.00 (0.00 to 0.00)	1.00 (0.00 to 0.00)
Women	1.32 (1.10 to 1.57)	1.32 (1.09 to 1.51)	1.33 (1.10 to 1.60)	1.60 (1.27 to 2.01)	1.62 (1.26 to 2.08)	1.63 (1.27 to 2.09)
Education (years)						
>9	1.00 (0.00 to 0.00)	1.00 (0.00 to 0.00)	1.00 (0.00 to 0.00)	1.00 (0.00 to 0.00)	1.00 (0.00 to 0.00)	1.00 (0.00 to 0.00)
≤9	1.31 (1.10 to 1.57)	1.33 (1.09 to 1.64)	1.31 (1.07 to 1.61)	1.51 (1.20 to 1.90)	1.68 (1.27 to 2.21)	1.59 (1.20 to 2.11)
Social class						
Non-manual	1.00 (0.00 to 0.00)	1.00 (0.00 to 0.00)	1.00 (0.00 to 0.00)	1.00 (0.00 to 0.00)	1.00 (0.00 to 0.00)	1.00 (0.00 to 0.00)
Manual	1.14 (0.96 to 1.36)	1.01 (0.83 to 1.23)	1.00 (0.82 to 1.23)	1.13 (0.91 to 1.40)	0.88 (0.68 to 1.14)	0.85 (0.54 to 1.10)
Number of chronic illness						
None	1.00 (0.00 to 0.00)	1.00 (0.00 to 0.00)	1.00 (0.00 to 0.00)	1.00 (0.00 to 0.00)	1.00 (0.00 to 0.00)	1.00 (0.00 to 0.00)
1	1.59 (1.27 to 2.00)	1.59 (1.25 to 2.02)	1.59 (1.25 to 2.02)	1.46 (1.08 to 1.99)	1.48 (1.06 to 2.05)	1.46 (1.05 to 2.03)
2+	1.83 (1.47 to 2.28)	1.81 (1.43 to 2.28)	1.82 (1.45 to 2.30)	2.07 (1.55 to 2.75)	2.07 (1.52 to 2.83)	2.06 (1.51 to 2.81)
*Area deprivation*						
Q1 (Least)	1.00 (0.00 to 0.00)		1.00 (0.00 to 0.00)	1.00 (0.00 to 0.00)		1.00 (0.00 to 0.00)
Q2	0.89 (0.67 to 1.20)		0.78 (0.58 to 1.05)	1.25 (0.81 to 1.92)		1.03 (0.66 to 1.61)
Q3	1.09 (0.82 to 1.45)		0.92 (0.68 to 1.24)	1.41 (0.93 to 2.14)		1.12 (0.72 to 1.75)
Q4 (Most)	1.41 (1.07 to 1.85)		1.09 (0.79 to 1.49)	2.03 (1.37 to 2.99)		1.52 (0.97 to 2.39)
*Natural environment*						
Q1 (Lowest)	1.00 (0.00 to 0.00)	1.00 (0.00 to 0.00)	1.00 (0.00 to 0.00)	1.00 (0.00 to 0.00)	1.00 (0.00 to 0.00)	1.00 (0.00 to 0.00)
Q2	0.79 (0.60 to 1.03)	0.75 (0.57 to 0.98)	0.76 (0.57 to 0.99)	0.77 (0.53 to 1.10)	0.71 (0.49 to 1.04)	0.75 (0.51 to 1.09)
Q3	0.86 (0.66 to 1.12)	0.88 (0.67 to 1.16)	0.93 (0.71 to 1.24)	0.76 (0.53 to 1.09)	0.78 (0.54 to 1.13)	0.88 (0.60 to 1.30)
Q4 (Highest)	0.58 (0.44 to 0.78)	0.62 (0.46 to 0.83)	0.67 (0.48 to 0.92)	0.52 (0.34 to 0.79)	0.55 (0.35 to 0.84)	0.66 (0.41 to 1.07)
p Value for trend	<0.01	<0.01	0.07	<0.01	0.01	0.17

Model 1: unadjusted; model 2: adjusted for all individual-level factors; model 3: adjusted for all individual-level factors and area deprivation.

In model 3, area deprivation was not strongly associated with clinical depression or anxiety symptoms, although the odds of subthreshold depression significantly increased from the least to most deprived areas (OR 1.98, 95% CI 1.34 to 2.93). Controlling for deprivation had only a small influence on the strength of association between these mental disorders and natural environment exposure (model 3) with a general trend of slight attenuation of the magnitude of relationships. After controlling for area deprivation, the strength of association between the natural environment and subthreshold depression was attenuated and the effect size in the highest quartile of natural environment reduced by about 20% (OR 0.66 vs 0.84) compared with the estimates in model 2.

## Discussion

### Main findings

This study found that a high exposure to green space and gardens in local areas was associated with lower odds of common mental disorders in later life after adjusting for individual sociodemographic factors and measures of comorbidity. Living in the highest quartile of neighbourhood natural environment provision was associated with a nearly 40% reduced odds compared with living in the lowest quartile. Controlling for area deprivation attenuated the strength of association for subthreshold depression but had limited influence on associations with anxiety symptoms and the co-occurrence of depressive and anxiety symptoms.

### Strengths and limitations

This study was able to utilise a large and well-characterised cohort of older people across heterogeneous areas of England. A structured interview using validated instruments to elicit information on psychiatric symptoms was used to measure depression and anxiety with consistent diagnostic standards across study centres. The CFAS interview also collected detailed information on comorbidities, and therefore their potential confounding effects could be taken into account in the analysis.

In terms of limitations, the study was cross-sectional, and therefore the associations observed cannot be assumed to be causal. The study population comprised survivors from the baseline interview 10 years earlier; individuals with more disadvantaged socioeconomic status and poor health status have been reported to have a higher risk of mental disorders and are more likely to be lost to follow-up, so findings might be slightly attenuated by this.^27^

Although the GMS-AGECAT provides a structured and systematic method to investigate mental disorders in later life, specific types of anxiety disorders such as panic disorder and phobias were not identified due to lack of a clinical diagnostic stage. Since the prevalence of clinical cases of anxiety disorders was low, this study focused on any level of anxiety symptoms. Nevertheless, although these subthreshold symptoms did not necessarily achieve clinical significance and severity, it is known that they can substantially affect the quality of life and well-being of older people.^28^ ^29^

The measure of exposure to the natural environment used was the amount of green space and private gardens in the geographical unit within each respondent lived. It may be that this spatial scale was not the most appropriate to define neighbourhood exposure. Further, no information was available on the quality and accessibility of local green space. No details were collected on time spent out of doors, and therefore the association with actual interactions with green spaces in communities could not be tested. Although the concept of ‘greenness’ might be different in urban and rural areas,^8^ ^30^ the rural population in CFAS was small (N=380, 15.7%) and skewed with 90% in the highest quartile of the natural environment exposure. It was therefore not possible to examine if associations with exposure to the natural environment differed between those in rural and urban areas. We did however conduct a subanalysis focusing solely on the urban population and found a somewhat stronger relationship between natural environment exposure, depressive and anxiety symptoms (see online supplementary table S1, supporting information).

### The natural environment and mental health in later life

The findings of this study suggest that a high exposure to natural environment in communities may be beneficial to mental health of older people in England. These findings also suggest a stronger association in older people compared with previous studies in younger age groups, which generally reported a small effect size of less than 10%.^6^ ^7^ Although the causal mechanisms linking natural environment exposure to mental disorders among older people could not be investigated in this cross-sectional study, some behavioural factors, such as physical activity, emotion and stress, have been considered to be associated with the exposure to green space in neighbourhoods. In the literature, psychological restoration and physical activity have been suggested to be two pathways to explain the positive influence of the natural environment on mental health.^31^ A high exposure to green space is thought to have direct effects on stress reduction or indirectly influence individual mental health through increasing outdoor activity.^32^ ^33^ A small number of existing studies have reported that the amount of green space or patterns of land use in local areas were associated with biomarkers related to stress and inflammation in middle age or older adults, although such information was unavailable to this study.^34–36^ In this older population aged 74 and above, the high prevalence of chronic conditions might also limit opportunities for physical activity, although how this may be moderated by the exposure to natural environments within which to be active is unknown.

The association between natural environment exposure and subthreshold depression was only somewhat attenuated after controlling for area deprivation. It is noteworthy that controlling for area deprivation attenuated associations with anxiety less than it did for depression. High exposure to natural environments in local areas may be particularly important to moderate feelings of unease, worry and fear. Our findings suggest a complex influence of multiple environmental factors and green space on depression symptoms in later life. The Indices of Multiple Deprivation examined include several compositional and contextual indicators such as unemployment, overcrowding, air pollution and crime, which have been associated with depression in existing studies.^37–39^ Further, a large population-based study in England has reported a high exposure to green space in local areas to be associated with lower levels of income-related health inequality.^40^ A combination of environmental (lack of natural environment, air pollution, crime) and individual factors (socioeconomic disadvantage, stressful life events, poor health status) might therefore act to increase the risk of depressive symptoms in later life.

### Implications in public health and clinical practice

This study indicates a potential positive influence of exposure to green space on mental health in later life. Although this study is cross-sectional in nature, the findings provide some indication that the provision of natural environments in neighbourhoods may be an effective public health intervention to maintain good mental health in older adults. Land use planners need to consider further ways in which to best support older people using local green space as promoting use of green space in older adults may help support healthy ageing. Ecotherapy, a wide range of programmes related to various activities in green environments, has been proposed as a potentially efficacious mechanism to moderate stress and depression, particularly when used as an adjunct to some type of formal therapy such as cognitive behaviour therapy.^41^ ^42^ Our findings suggest clinical practice and primary care settings could play a role in this potential promotion of well-being in later life by signposting patients to opportunities such as the local availability of health walks which provide group-based walking opportunities in natural settings.

### Future research directions

Although potential mechanisms that may explain the beneficial influence of the natural environment on individual mental health have been proposed, actual interactions between older people and green space need to be further investigated. Our research provides circumstantial support for programmes encouraging exposure to natural environments in local areas, but longitudinal studies involving the study of patterns of migration and the impact of changes in provision of natural environments will be avenues for creating a more rigorous evidence base. Since mental health benefits of physical activity have been shown to be greater when activity is performed in green settings,^43–45^ more detailed information on the usage of green space in older populations could be provided by qualitative studies that investigate various subgroups such as gender, ethnicity and socioeconomic status, and also from quantitative studies that employ technologies such as global positioning systems and audit tools to track activity patterns.^46^ ^47^ Different types and qualities of green space might also have differential influences on the mental health of older people, and a better understanding of how green space characteristics might influence their use and cumulative mental health benefits through outdoor activity is needed.

## References

[1] Lachowycz K, Jones AP Towards a better understanding of the relationship between greenspace and health: development of a theoretical framework. Landscape Urban Plann 2013;118:62–9. 10.1016/j.landurbplan.2012.10.012

[2] Croucher K, Myers L, Jones R Health and the physical characteristics of urban neighbourhoods: a critical literature review. 2008 http://www.gcph.co.uk/assets/0000/0447/Health_and_the_Physical_Characteristics_of_Urban_Neighbourhoods.pdf

[3] Greenspace Scotland. Greenspace Scotland research report: the links between greenspace and health: a critical literature review. 2008 http://www.york.ac.uk/media/chp/documents/2008/greenspace2008.pdf

[4] Power A, Davis J, Plant P Strategic review of health inequalities in England post-2010 Task Group 4: the built environment and health inequalities. 2009 http://www.instituteofhealthequity.org/projects/built-environment-marmot-review-task-group-report

[5] World Health Organisation. Hidden cities: unmasking and overcoming health inequalities in urban settings 2010 http://www.who.int/kobe_centre/publications/hidden_cities2010/en/

[6] Astell-Burt T, Feng X, Kolt GS Mental health benefits of neighbourhood green space are stronger among physically active adults in middle-to-older age: evidence from 260,061 Australians. Prev Med 2013;57:601–6. 10.1016/j.ypmed.2013.08.017 23994648

[7] Nutsford D, Pearson AL, Kingham S An ecological study investigating the association between access to urban green space and mental health. Public Health 2013;127:1005–11. 10.1016/j.puhe.2013.08.016 24262442

[8] Maas J, Verheij RA, Groenewegen PP Green space, urbanity, and health: how strong is the relation? J Epidemiol Community Health 2006;60:587–92. 10.1136/jech.2005.043125 16790830 PMC2566234

[9] Alcock I, White MP, Wheeler BW Longitudinal effects on mental health of moving to greener and less green urban areas. Environ Sci Technol 2013;48:1247–55. 10.1021/es403688w 24320055

[10] Bixby H, Hodgson S, Fortunato L Associations between green space and health in English cities: an ecological, cross-sectional study. PLoS ONE 2015;10:e0119495 10.1371/journal.pone.0119495 25775020 PMC4361406

[11] Taylor MS, Wheeler BW, White MP Research note: urban street tree density and antidepressant prescription rates—a cross-sectional study in London, UK. Landscape Urban Plann 2015;136:174–9. 10.1016/j.landurbplan.2014.12.005

[12] Macintyre S, Ellaway A, Cummins S Place effects on health: how can we conceptualise, operationalise and measure them? Soc Sci Med 2002;55:125–39. 10.1016/S0277-9536(01)00214-3 12137182

[13] Davis MG, Fox KR, Hillsdon M Objectively measured physical activity in a diverse sample of older urban UK adults. Med Sci Sports Exerc 2011;43:647–54. 10.1249/MSS.0b013e3181f36196 20689449

[14] Pleson E, Nieuwendyk L, Lee K Understanding older adults’ usage of community green spaces in Taipei, Taiwan. Int J Environ Res Public Health 2014;11:1444–64. 10.3390/ijerph110201444 24473116 PMC3945547

[15] Lui CW, Everingham JA, Warburton J What makes a community age-friendly: a review of international literature. Australas J Ageing 2009;28:116–21. 10.1111/j.1741-6612.2009.00355.x 19845650

[16] Sugiyama T, Thompson CW, Alves S Associations between neighborhood open space attributes and quality of life for older people in Britain. Environ Behav 2009;41:3–21. 10.1177/0013916507311688

[17] Sarkar C, Gallacher J, Webster C Urban built environment configuration and psychological distress in older men: results from the Caerphilly study. BMC Public Health 2013;13:695 10.1186/1471-2458-13-695 23898839 PMC3735426

[18] Brayne C, McCracken C, Matthews FE Cohort profile: the Medical Research Council Cognitive Function and Ageing Study (CFAS). Int J Epidemiol 2006;35:1140–5. 10.1093/ije/dyl199 16980700

[19] Copeland JR, Dewey ME, Griffiths-Jones HM A computerized psychiatric diagnostic system and case nomenclature for elderly subjects: GMS and AGECAT. Psychol Med 1986;16:89–99. 10.1017/S0033291700057779 3515380

[20] Kvaal K, McDougall FA, Brayne C Co-occurrence of anxiety and depressive disorders in a community sample of older people: results from the MRC CFAS (Medical Research Council Cognitive Function and Ageing Study). Int J Geriatr Psychiatry 2008;23:229–37. 10.1002/gps.1867 17631679

[21] McDougall FA, Matthews FE, Kvaal K Prevalence and symptomatology of depression in older people living in institutions in England and Wales. Age Ageing 2007;36:562–8. 10.1093/ageing/afm111 17913759

[22] Office for National Statistics. Standard Occupational Classification, 1990 http://www.ons.gov.uk/ons/guide-method/classifications/archived-standard-classifications/soc-and-sec-archive/index.html

[23] Huang CQ, Dong BR, Lu ZC Chronic diseases and risk for depression in old age: a meta-analysis of published literature. Ageing Res Rev 2010;9:131–41. 10.1016/j.arr.2009.05.005 19524072

[24] Office for National Statistics. National Statistics Postcode Products. http://www.ons.gov.uk/ons/guide-method/geography/products/postcode-directories/-nspp-/index.html

[25] Office for National Statistics. Neighbourhood Statistics. http://www.neighbourhood.statistics.gov.uk

[26] Neighbourhood Renew Unit. The English Indices of Deprivation 2004 (Revised). Office of the Deputy Prime Minister, 2004.

[27] Matthews F, Chatfield M, Freeman C Attrition and bias in the MRC cognitive function and ageing study: an epidemiological investigation. BMC Public Health 2004;4:12 10.1186/1471-2458-4-12 15113437 PMC419705

[28] Vink D, Aartsen MJ, Schoevers RA Risk factors for anxiety and depression in the elderly: a review. J Affect Disord 2008;106:29–44. 10.1016/j.jad.2007.06.005 17707515

[29] Prina AM, Ferri CP, Guerra M Prevalence of anxiety and its correlates among older adults in Latin America, India and China: cross-cultural study. Br J Psychiatry 2011;199:485–91. 10.1192/bjp.bp.110.083915 22016438 PMC3227807

[30] Mitchell R, Popham F Greenspace, urbanity and health: relationships in England. J Epidemiol Community Health 2007;61:681–3. 10.1136/jech.2006.053553 17630365 PMC2652991

[31] Hartig T Green space, psychological restoration, and health inequality. Lancet 2008;372:1614–15. 10.1016/S0140-6736(08)61669-4 18994650

[32] Rosso AL, Auchincloss AH, Michael YL The urban built environment and mobility in older adults: a comprehensive review. J Aging Res 2011;2011:816106 10.4061/2011/816106 21766033 PMC3134204

[33] Van Cauwenberg J, De Bourdeaudhuij I, De Meester F Relationship between the physical environment and physical activity in older adults: a systematic review. Health Place 2011;17:458–69. 10.1016/j.healthplace.2010.11.010 21257333

[34] van den Berg AE, Maas J, Verheij RA Green space as a buffer between stressful life events and health. Soc Sci Med 2010;70:1203–10. 10.1016/j.socscimed.2010.01.002 20163905

[35] Roe JJ, Thompson CW, Aspinall PA Green space and stress: evidence from cortisol measures in deprived urban communities. Int J Environ Res Public Health 2013;10:4086–103. 10.3390/ijerph10094086 24002726 PMC3799530

[36] Woo J, Tang N, Suen E Green space, psychological restoration, and telomere length. Lancet 2009;373:299–300. 10.1016/S0140-6736(09)60094-5 19167568

[37] Wight RG, Aneshensel CS, Barrett C Urban neighbourhood unemployment history and depressive symptoms over time among late middle age and older adults. J Epidemiol Community Health 2013;67:153–8. 10.1136/jech-2012-201537 22918896 PMC3681821

[38] Wilson-Genderson M, Pruchno R Effects of neighborhood violence and perceptions of neighborhood safety on depressive symptoms of older adults. Soc Sci Med 2013;85:43–9. 10.1016/j.socscimed.2013.02.028 23540365

[39] Julien D, Richard L, Gauvin L Neighborhood characteristics and depressive mood among older adults: an integrative review. Int Psychogeriatr 2012;24:1207–25. 10.1017/S1041610211002894 22300529

[40] Mitchell R, Popham F Effect of exposure to natural environment on health inequalities: an observational population study. Lancet 2008;372:1655–60. 10.1016/S0140-6736(08)61689-X 18994663

[41] Mind for Better Mental Health. Make sense of ecotherapy 2013 http://www.mind.org.uk/media/311422/making-sense-of-ecotherapy-2013.pdf

[42] Stevens P Embedment in the environment: a new paradigm for well-being? Perspect Public Health 2010;130:265–9. 10.1177/1757913910384047 21213562

[43] Thompson Coon J, Boddy K, Stein K Does participating in physical activity in outdoor natural environments have a greater effect on physical and mental wellbeing than physical activity indoors? A systematic review. Environ Sci Technol 2011;45:1761–72. 10.1021/es102947t 21291246

[44] Pretty J, Peacock J, Sellens M The mental and physical health outcomes of green exercise. Int J Environ Health Res 2005;15:319–37. 10.1080/09603120500155963 16416750

[45] Bodin M, Hartig T Does the outdoor environment matter for psychological restoration gained through running? Psychol Sport Exerc 2003;4:141 10.1016/S1469–0292(01)00038–3

[46] Coombes E, van Sluijs E, Jones A Is environmental setting associated with the intensity and duration of children's physical activity? Findings from the SPEEDY GPS study. Health Place 2013;20:62–5. 10.1016/j.healthplace.2012.11.008 23376730 PMC3591252

[47] Hillsdon M, Panter J, Foster C The relationship between access and quality of urban green space with population physical activity. Public Health 2006;120:1127–32. 10.1016/j.puhe.2006.10.007 17067646

